# Wherever in the world you find nurses, you will find leaders

**DOI:** 10.1590/1518-8345.0000.3181

**Published:** 2019-05-16

**Authors:** Annette Kennedy

**Affiliations:** 1 International Council of Nurses, Genebra, Suíça.


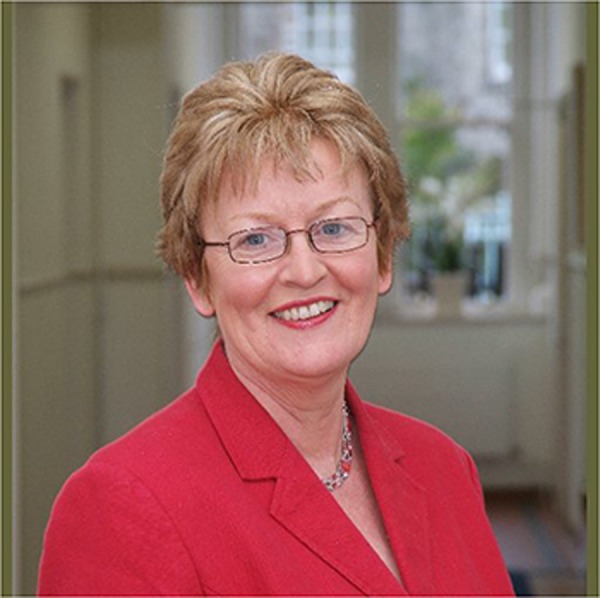


Nurses lead their colleagues and other professionals, they lead their teams, and they lead their patients to better health and more productive, healthier lifestyles.

But nurses could have an even greater impact on the world’s health if they were given more opportunities to work to their maximum capabilities, and greater influence in making decisions about health, social and economic policies.

The International Council of Nurses (ICN) is a federation of more than 130 National Nursing Associations that represents the more than 20 million nurses worldwide.

As ICN President, I am thrilled that the Nursing Now campaign, run in collaboration with ICN and the World Health Organization (WHO), is being adopted all around the world, including in South America.

Nursing Now is a three-year campaign that is intended to improve health by raising the profile and status of nursing worldwide.

It aims to empower nurses to help tackle 21st Century health challenges and maximise their contribution to achieving the goal of Universal Health Coverage.

Nursing Now focuses on:

- Ensuring that nurses and midwives have a more prominent voice in health policy-making.
- Encouraging greater investment in the nursing workforce.
- Recruiting more nurses into leadership positions.
- Conducting research that helps determine where nurses can have the greatest impact.
- Sharing best nursing practices.

At ICN we have always taken leadership seriously: in fact, our first leadership workshop took place in 1993 in Caracas, Venezuela.

That workshop was a forerunner to our current highly successful and influential leadership programmes, the Global Nursing Leadership InstituteTM (GNLI) and Leadership for ChangeTM (LFC).

Nurses know that policies and politics profoundly affect the health of populations locally, regionally and internationally, and that they shape the practice of nursing and the environments that nurses work in.

## Leadership for change

The ICN’s LFC programme works on the needs of nurses in specific countries. It provides them with the leadership skills they require to implement organisational change for the purpose of improving nursing practice and achieving better health outcomes.

The LFC programme provides participants with opportunities to develop their understanding of global health challenges, obtain insight into international leadership styles, and be exposed to and analyse change management in the context of health system redesign and transformation, and health and social policy.

## Global Nursing Leadership Institute

The GNLI programme is designed to take nurses who are already in the most senior positions in the profession and boost their ability to influence policymaking at the very highest levels of organisations and governments.

Each year, 30 of the world’s top nurses go through a rigorous development programme to increase their understanding of how to influence policy, improve their understanding of who their key stakeholders are, create clear policy messages that are effective in bringing about positive change and develop their skills in negotiating at the highest levels of government.

Each programme includes a week-long residential workshop in Geneva, which is highly interactive and enables participants to practice the new skills they have on experts from around the world.

It is true that leadership courses are not for every nurse, but people who take part in such courses end up sharing their experiences with their colleagues when they return home, and disseminating their knowledge far and wide.

It is heart-warming to see nurses from different parts of the world stepping up to the highest levels and making the case for nursing.

I want to congratulate Brazil for being the latest country to adopt Nursing Now, and to encourage others to follow in their footsteps.

